# Molecular Subtypes of Vulvar Squamous Cell Carcinoma: The Significance of HPV-Independent/p53 Wild Type

**DOI:** 10.3390/cancers16244216

**Published:** 2024-12-18

**Authors:** Lars-Christian Horn, Christine E. Brambs, Blake Gilks, Lien Hoang, Naveena Singh, Grit Gesine Ruth Hiller, Kathrin Hering, Jessica N. McAlpine, Amy Jamieson, Mona Alfaraidi, Bahriye Aktas, Nadja Dornhöfer, Anne Kathrin Höhn

**Affiliations:** 1 Division of Gynecologic, Breast and Perinatal Pathology, Institute of Pathology, University Hospital Leipzig, D-04103 Leipzig, Germany; ruth.hiller@uniklinik-leipzig.de (G.G.R.H.); annekathrin.hoehn@medizin.uni-leipzig.de (A.K.H.); 2 Department of Obstetrics and Gynecology, Kantonsspital Luzern, 6004 Luzern, Switzerland; christine.brambs@luks.ch; 3 Department of Anatomical Pathology, Vancouver General Hospital, The University of British Columbia, Vancouver, BC V5Z 1M9, Canada; blake.gilks@vch.ca (B.G.); lien.hoang@vch.ca (L.H.); naveena.singh@vch.ca (N.S.); mona.alfaraidi@vch.ca (M.A.); 4 Department of Radiotherapy and Radiation Oncology, University Hospital Leipzig, D-04103 Leipzig, Germany; kathrin.hering@medizin.uni-leipzig.de; 5 Division of Gynecologic Oncology, Department of Obstetrics and Gynecology, The University of British Columbia, Vancouver, BC V5Z 1M9, Canada; jessica.mcalpine@vch.ca (J.N.M.); amy.jamieson@vch.ca (A.J.); 6 Prince Sultan Military Medical City, Riyah 12233, Saudi Arabia; 7 Division of Gynecologic Oncology, Department of Obstetrics and Gynecology, Institute of Trier, University Hospital Leipzig, D-04103 Leipzig, Germany; bahriye.aktas@medizin.uni-leipzig.de (B.A.); nadja.dornhoefer@medizin.uni-leipzig.de (N.D.)

**Keywords:** vulvar cancer, squamous cell carcinoma, p53, p16, human papillomavirus, molecular, classification, prognosis, radiation therapy, surgery

## Abstract

Vulvar carcinoma is a rare disease, meeting the criteria for a “rare cancer”, but its incidence is increasing, especially in women <60 years of age. Squamous cell carcinoma (VSCC) accounts for the overwhelming majority of vulvar carcinomas and is the focus of this review. As with many cancers, the increased understanding of molecular events during tumorigenesis has led to the emergence of the molecular subclassification of VSCC, which is subclassified into tumors that arise secondary to high-risk human papillomavirus infection (HPV-associated, or HPVa) and those that arise independently of HPV (HPVi), most commonly in the setting of a chronic inflammatory condition of the vulvar skin. This latter group of HPVi VSCC arises in most cases secondary to mutations in *TP53*, but recently, attention has focused on the uncommon *TP53* wild-type HPVi VSCC. These three molecular subtypes of VSCC (HPVa, HPVi p53 abnormal, and HPVi p53 wild type), as well as their precursor lesions, cannot be diagnosed based on a routine histopathological examination or immunostaining for p53 and p16 as surrogate markers for *TP53* mutation and high-risk HPV infection, respectively, are required. The molecular subtyping of VSCC shows high reproducibility and provides important prognostic information. HPVa VSCC has the most favorable prognosis, while HPVi VSCC with *TP53* mutations (p53abn) has the worst prognosis, and HPVi VSCC with wild-type *TP53* (p53wt) has an intermediate prognosis. In this review, we discuss the evidence supporting this molecular subclassification and its implications for the diagnosis and treatment of VSCC and its precursors.

## Simple Summary

The distinct histopathogenetic pathways of vulvar squamous cell neoplasia have led to a shift from a morphology-based to an etiology/molecular-based histopathologic classification, adopted in the most recent WHO Classification of Female Genital Tumours. There are two main categories: HPV-associated (HPVa) and HPV-independent (HPVi) vulvar squamous neoplasia. Most HPVi VSCCs have an underlying *TP53* mutation, but attention has recently focused on the uncommon third molecular subtype: *TP53* wild-type HPVi VSCC. We review this shift to the etiology/molecular-based classification of VSCC, emphasizing the diagnostic and treatment implications of this change and highlighting the third molecular subtype.

## 1. Introduction

Vulvar cancer (VC) is an uncommon disease [1] and meets the criteria for a “rare cancer” according to the definition of the European Society for Medical Oncology, i.e., cancer with an incidence of <6 per 100,000 per year [2,3]. VC accounts for approximately 5.6% of all gynecologic malignancies, with an estimated 6470 new cases in 2023 in the US [4]. An Australian study reported an 84% increase in VC incidence from 1982 to 1984 to 2007–2009 in women younger than 60 years, with no change in incidence in women 60+ years [5,6]. Comparing the cancer registry data from 13 high-income countries over a 20-year period, Kang et al. [6] reported an overall increase of 4.6%, with an increase of 11.6% in women < 60 years of age but no change in women over 60 years of age.

There has been no recent change in the overall survival of vulvar cancer patients [7]. Regardless of tumor stage and treatment approaches, an analysis of the SEER datasets between 2000 and 2007 reported a 5-year overall survival of 60.6% (45.7% at 10 years and 33.1% at 15 years) [8]. The vast majority of VCs are squamous cell carcinomas (VSCCs) [8,9,10]. Within this histologic subtype, the most important development in the last 10 years has been the recognition of distinct histopathogenetic pathways [9,10,11,12]; these pathways are associated with distinct precursor lesions, different responses to treatment, and different outcomes [11,13,14,15,16,17,18,19,20,21,22]. Here, we summarize the clinicopathologic features of the different molecular subtypes of VSCC, including their impact on diagnosis and treatment.

## 2. Molecular Subtypes of VSCC

**Figure 1:**
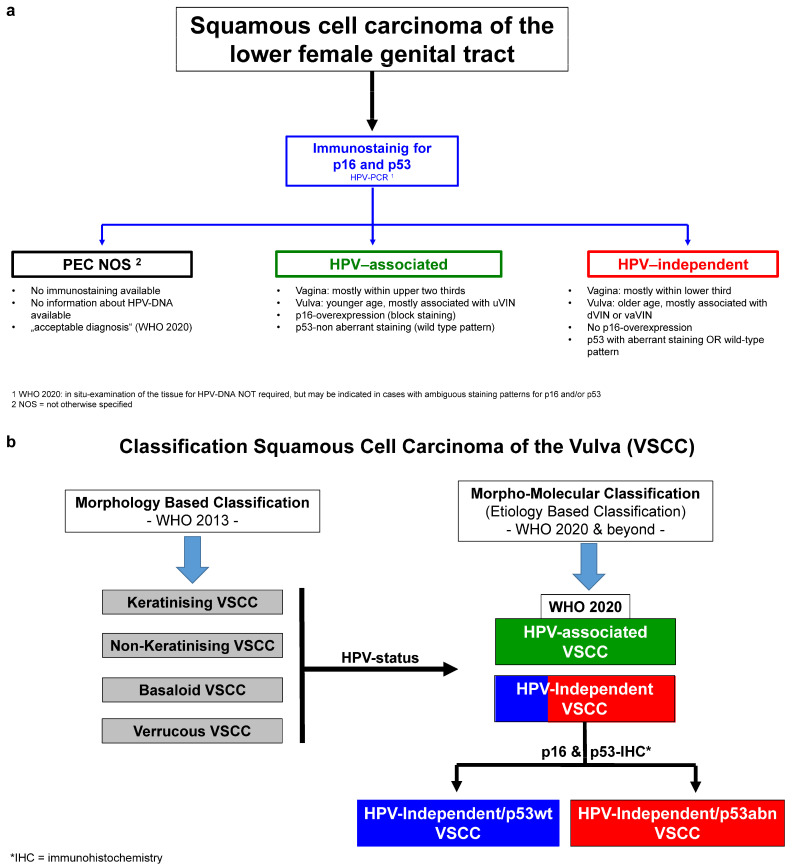
(**a**) Subdivision of squamous cell carcinomas of the lower female genital tract based on HPV association according to the recommendations of the WHO classification 2020 [28]; (**b**): Classification of VSCC from morphology-based to morpho-molecular classification, depending on the etiology of the disease, using p16 and p53 immunohistochemistry [10,12,14,20].

Although it has been recognized since the early 1990s that VSCC can either be associated with high-risk human papillomavirus infection (HPVa) or independent of HPV (HPVi) [23,24], it has only recently been appreciated that VSCC and squamous cell carcinomas at other body sites, e.g., the oropharynx [25,26,27], differ significantly from HPVi squamous cell carcinomas arising at the same body site. It is only in the fifth edition of the WHO Classification of Female Genital Tumours [28] that VSCC and its precursor lesions have been subclassified as HPVi or HPVa (Figure 1a; [28]).

The move from the previous morphology-based classification of VSCC to an etiology/molecular-based classification (Figure 1b) allows for a more precise prognosis characterization [10,12,14,18]; the same also occurred for cervical squamous cell carcinomas and adenocarcinomas (Figure 1a,b; [28]). This change was subsequently adopted by the International Collaboration on Cancer Reporting (ICCR; [29]) and the College of American Pathologists [29,30], as well as by the most recent update of the guidelines of the European Society of Gynaecological Oncology for the management of patients with vulvar cancer [31].

The molecular subclassification of VSCC defines at least two distinct subtypes (for details and diagnostic approaches (please see Figure 1b below)). Briefly, the first most common and aggressive molecular subtype is HPV-independent and is associated with a *TP53* mutation, consequently resulting in an aberrant p53 protein expression on immunohistochemistry. The second less common and less aggressive subtype is HPV-associated and is based on an association with high-risk HPV infection, showing a better prognosis and p16 protein overexpression [9,10,14,16].

This molecular subclassification has been implemented at sites outside the female genital tract where there is HPVa squamous cell carcinoma, e.g., in the penis [32], as well as in head and neck cancers [27,33,34,35]. While most HPVi VSCCs have mutations in *TP53*, associated with a mutant-pattern expression of the p53 protein on immunostaining, Sand et al. [36] evaluated p16 and p53 expression in 18 studies on VSCC and found that 54% of the tumors were HPVi with mutant-pattern p53 expression and that 38% were HPVa with immunohistochemical p16 overexpression; however, 8% of VSCCs were neither p16-negative with mutant-pattern p53 nor p16-positive with wild-type 53, thus pointing to a third molecular subtype of VSCC, namely, the HPVi (p16-negative) and p53 wild type.

Nooji et al. [37] were the first to report on this third molecular subtype of VSCC. They performed a targeted NGS analysis of VSCC and its precursors and found a higher frequency of *NOTCH1* and *HRAS* mutations in HPVi p53wt VSCC than in HPVi p53abn VSCC. They, therefore, suggested that *NOTCH1* and *HRAS* mutations may act as drivers of mutational events independent of *TP53*, and this was later supported by another study [15]. Furthermore, high rates (up to 71%) of *NOTCH1* mutations have been reported to occur in penile cancer, a male tumor with many similarities to VSCC [38].

**Figure 2:**
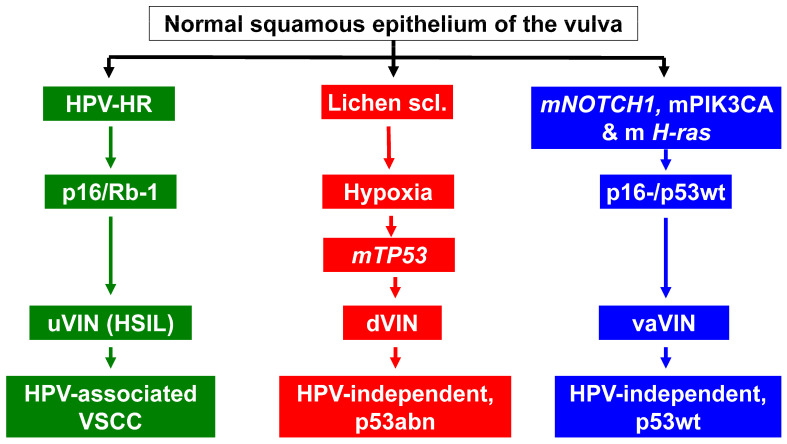
Summary of the recently described three-tiered molecular classification of VSCC and its precursors (please see text) (uVIN = usual VIN; dVIN = differentiated VIN; vaVIN = verrucous VIN).

The prognosis of the three molecular subtypes of VSCC (HPVa, HPVi p53abn, and HPVi p53wt) shows that HPVa VSCC has the most favorable prognosis, HPVi p53abn has the worst prognosis, and HPVi p53wt has an intermediate prognosis [12,18,37,39]; thus, molecular analyses have been performed [15,37]. As a result, there is growing acceptance of the routine classification of VSCC and its precursors into one of these three molecular subtypes (see Figure 2).

## 3. Diagnosis of Molecular Subtypes of VSCC

The molecular subclassification of VSCC is based on the routine use of molecular markers, specifically immunostaining for both p53 and p16 in all cases. Ambiguous staining results may require additional in situ analyses of HPV and/or *TP53* sequencing.

The immunohistochemical evaluation of p53 is performed as a pattern-based analysis rather than as a form of simple positive or negative staining. The different staining patterns are summarized in Table 1, and when interpreted according to these recommendations, they show a strong correlation with *TP53* mutational status.

Immunohistochemical staining for p16, as a surrogate marker of high-risk HPV infection, has been recommended as part of the routine stratification of VSCC by the International Collaboration on Cancer Reporting (ICCR), the International Federation of Gynecology and Obstetrics (FIGO), and the WHO [1,14,17,28,29,41,42].

**Figure 3:**
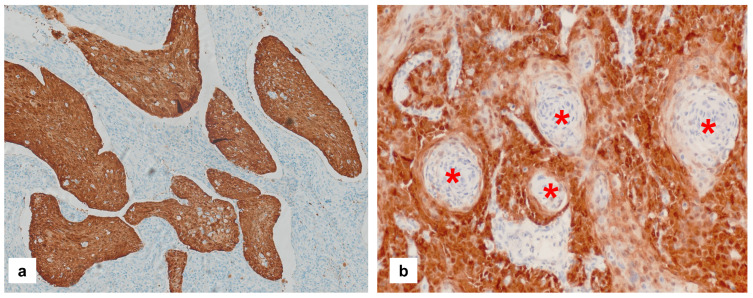
Patterns of p16 overexpression in non-keratinizing (**a**) and keratinizing (**b**) VSCC, so-called block-like staining. Note that the keratinized areas stain negative for a p16 pattern (asterisk; see text).

In vulvar intraepithelial neoplasia (VIN) lesions, p16 is considered overexpressed when there is block-like staining, i.e., a strong nuclear and cytoplasmic staining of all lesional cells from the basal layer upwards through at least one-third of the epithelial thickness [43,44]. In invasive or metastatic VSCC, p16 overexpression is recognized when moderate or intense nuclear and cytoplasmic staining is detected in 70% or more of the tumor cells [9,44]. This interpretation of p16 staining in invasive carcinoma is based on the recommendations for head and neck squamous cell carcinoma by the American Society of Clinical Oncology and the College of American Pathologists [26]. The foci of keratinization in VSCC should be excluded from consideration (Figure 3, [9]).

## 4. Accuracy of Molecular Subclassification of VSCC

Given its concordance with in situ detection methods for HPV of up to 100%, p16 overexpression is considered a reliable surrogate marker for high-risk HPV infection [39,41,45,46]. The abovementioned p16 immunostaining has been reported to have a sensitivity of 97.4% to 100% and a specificity of 96.6% to 98.7% in the assessment of HPV status [14,42]. Very rarely, the complete absence of p16 overexpression may be seen in HPVa carcinomas of the lower female genital tract [47]; this may be caused by a loss of heterozygosity [48], rarely by point mutations of the p16 gene or the silencing of the gene by promotor hypermethylation [49].

In VSCC, p53 immunostaining interpretation has to be performed using a pattern-based analysis, defining two wild-type and four aberrant staining patterns (see Table 1). Aberrant p53 patterns show a strong correlation with *TP53* sequencing analyses in VSCC (95% agreement) [16]. Another study reported a 97% accuracy of p53 immunostaining in the prediction of *TP53* mutations in VSCC [50].

It is important to note that molecular classification is not possible based on hematoxylin and eosin-stained slides [17,19,42,51]. Therefore, the use of p16 and p53 immunohistochemistry is necessary for the correct molecular subtyping of VSCC. Using these two immunostains to diagnose the three different molecular subtypes (Figure 2), Yang et al. [14] reported that 95.6% (215/225) of VSCC could be readily classified as one of the three subtypes by using p16 and p53 immunostaining alone. Thompson et al. [20] demonstrated a good interobserver correlation for the immunohistochemical classification of VSCC into one of three molecular subtypes using p16 and p53 immunohistochemistry, with a kappa value of 0.74. In cases with ambiguous immunohistochemical staining results, it is sometimes necessary to examine more than one area of the tumor and more than one tissue block [14]. Importantly, immunohistochemistry for p53 and p16 is inexpensive and readily available; it represents a robust method with a standardized technical workup, and the results are usually available within 24 hours [17,19].

**Figure 4:**
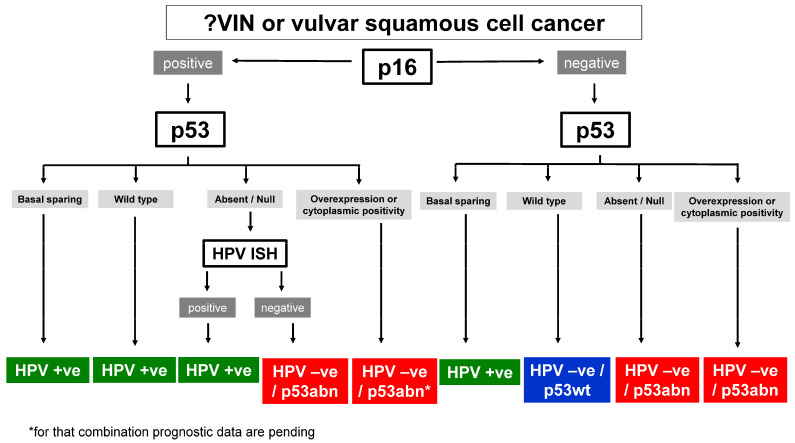
An algorithmic approach for the three-tiered molecular subclassification of VSCC using p16 and p53 immunohistochemistry [14]. ISH = in situ hybridization.

In cases with ambiguous staining results (<5% of cases), an additional molecular evaluation may be required [14]. An algorithmic approach for use in further molecular testing (in situ hybridization or sequencing), as suggested by Yang et al. [14], is summarized in Figure 4.

**Figure 5:**
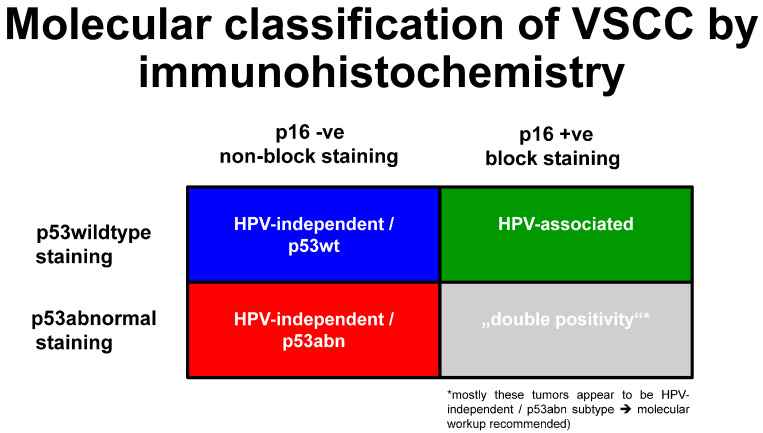
Immunohistochemical classification of VSCC into its molecular subtypes (for details, please see the text and Figure 4 and Figure 6 and Table 1).

One final consideration in the molecular subtyping of VSCC is that HPV high-risk-negative p53abn VSCC may show both an immunohistochemical overexpression of p16 and a mutant-pattern p53 expression, which is so-called *double positivity*. The frequency of these cases ranges from 0% [22,52] to 4.2% [14] and 6.8% [53] and may be underestimated at this time. With regard to the mechanism of double positivity, p16 is frequently overexpressed in high-grade serous ovarian cancer, and <1% of these tumors harbor a *CDKN2A* mutation, so p16 overexpression is thought to be secondary to dysregulation and to underlying p53 and/or Rb mutations [54]. In VSCC, Yang et al. [14] reported a *CDKN2A* (frameshift) mutation in two out of five of their double-positive cases. Clearly, the reason for double-positive staining in VSCC requires further research, but the small number of cases reported to date suggests that the combination of p16 positivity and mutant-pattern p53 staining is usually seen in HPVi p53abn VSCC. The molecular subclassification of VSCC is summarized in Figure 5.

## 5. Prognostic Impact of Molecular Subtyping

It is now accepted that the HPV status of VSCC has a highly significant impact on prognosis [17,19,20,36,52,53,55,56], and it has been the subject of two meta-analyses. Cao et al. [57] reported a higher overall survival (RR = 0.53, (95% CI [0.35–0.80]; *p* = 0.003) for HPVa/p16-positive tumors. Sand et al. [36] reported a pooled hazard ratio of 0.40 (95% CI [0.29–0.55]) for p16-positive VSCC and 1.81 (95% CI [1.22–2.68]) for tumors with mutant-pattern p53 expression.

McAlpine et al. [19] reported significantly worse survival in HPVi and p53-driven VSCC in the era following the move away from en bloc radical vulvectomy. Their study (*n* = 197) compared two surgical approaches: radical vulvectomy with en bloc resection of the vulva and groins (called long-horn excision; group 1) versus a localized radical vulvectomy with separate incisions for the vulva and both groins (so-called triple incision; group 2). HPVa tumors were shown to have a superior progression-free (HR 0.17; *p* < 0.001), disease-specific (HR 0.08; *p* < 0.001), and overall survival (HR 0.24; *p* < 0.001) in the group treated with less radical surgery. In contrast, HPV status had no prognostic relevance in the group treated with the more radical surgical approach. Furthermore, 30–40% of the patients in both groups received adjuvant radiation therapy, with no reported inter-group differences in the radiation technique. In the radical surgical technique of a vulvar field resection (VFR), the extent of the resection (partial, total, or extended) and regional lymph node sampling is based on the understanding of embryonic and fetal development, aiming to resect the ontogenetic cancer field. Early evidence showed that VFR without adjuvant radiotherapy compares favorably to standard multimodal treatment [58]. A study by Thompson et al. [20] reported equivalent outcomes in HPVa and HPVi p53abn VSCCs treated with the VFR-based surgical approach. These results are consistent with those of the study by McAlpine et al. [19], leading to the hypothesis that a radical primary resection leads to an improved outcome for patients with a high-risk molecular subtype of VSCC, i.e., HPVi p53abn. Ultimately, however, a prospective clinical trial of different surgical approaches is needed to adequately test the hypotheses that more radical surgery leads to an improved outcome for patients with HPVi VSCC and that equivalently favorable outcomes can be achieved in select cases of younger patients with HPVa VSCC through less aggressive surgery with adjuvant radiotherapy.

With regard to adjuvant radiotherapy, Dohopolski et al. [59] examined 39 patients with adjuvant external-beam (chemo-) RT and reported an in-field relapse rate of 72.2% for HPVi and 59.1% for HPVa tumors (*p* = 0.062), as well as a five-year progression-free survival of 22.1% versus 63.5% (*p* = 0.072). Two other studies mainly included patients treated with adjuvant radiation only: Yap et al. ([60]; 26/40 with adjuvant RT only) described a locoregional relapse rate of 81.2% for HPVi versus 15.4% for HPVa VSCC (*p* = 0.003), and similar results were reported by Lee et al. ([61]; 28/57 with adjuvant RT only), with a local recurrence rate of 75% versus 19% (*p* < 0.01). In a study by Lindell et al. [62], 24 of 75 patients underwent adjuvant RT, showing a significantly better recurrence-free, disease-specific, and overall survival in those with HPVa tumors (*p* ≤ 0.03). In a cohort of 73 patients with a median tumor size of 5.0 cm treated initially with radiotherapy, a complete clinical response rate of 63.6% was reported in HPVa versus 35.0% in HPVi VSCC [53]. In patients treated with a neoadjuvant treatment approach (*n* = 63), the pathologic complete response rate was 53.8% versus 31.4% (*p* = 0.067, HPVa/p16 + ve versus HPVi/p16-ve, respectively). Proctor et al. [55] reported a higher complete clinical response rate in 48 patients with definitive (chemo-) radiation for HPVa (72.2%) versus 42.9% in HPVi VSCC. A case of HPVa VSCC with a dramatic clinical response to chemoradiation is illustrated in Figure 7. It should be noted that these studies, while strongly suggesting a difference in radiosensitivity between HPVa and HPVi VSCC, are retrospective, and there are no randomized prospective studies of VSCC where the molecular subtype is determined.

## 6. HPV-Independent, p53 Wild-Type VSCC: Clinical Features

Most studies exploring the prognostic impact of molecular subtypes in VSCC have not subdivided the HPVi VSCC into p53abn and p53wt, so the data comparing all three molecular subtypes are still limited. Some studies excluded HPVi p53wt tumors from the analyses because of their rarity [20]. Both Hinten et al. [11] and Barlow et al. [18] reported details of the HPVi p53wt tumor molecular subtype only in the Supplementary Material and not in the main analyses.

As previously noted, HPVi p53wt is the least common of the three subtypes (see Table 2).

To illustrate the clinicopathologic findings of the three-tiered molecular classification of VSCC, Table 3 summarizes the results of two recent large studies analyzing *n* = 413 cases [12] and *n* = 411 cases [39], respectively.

Nooji et al. [37] evaluated a series of 236 VSCCs and reported a three times higher rate of local recurrence in HPVi p53wt and a four times higher recurrence rate in HPVi p53abn VSCC than in HPVa cancers (*p* = 0.044). A study by Kortekaas et al. [12] reported better five-year overall survival in HPVa VSCC than in HPVi p53wt (HR 2.16; *p* = 0.049) and HPVi p53abn VSCC (HR 3.43; *p* < 0.001). Comparing the two HPVi molecular subtypes, p53wt VSCC also had a better five-year overall survival than p53abn tumors (HR 0.63; *p* = 0.06), and p53wt tumors showed a longer recurrence-free period than p53abn VSCC (HR 0.43; *p* = 0.003; [12]). In a multivariate analysis, the three-tiered molecular classification of VSCC significantly correlated with the risk of recurrence irrespective of other clinicopathologic factors (e.g., tumor stage, tumor size, the depth of invasion, and inguinal lymph node involvement). Interestingly, recurrent disease occurred locally in all three molecular subtypes.

Table 4 summarizes the prognostic data obtained from the literature for the three-tiered molecular classification of VSCC.

## 7. Future Directions

Different targeted treatment approaches have been reported for VSCC (e.g., targeting EGFR (erlotinib), VEGF (bevacizumab), and PD-L1 (pembrolizumab; [64])). However, the results of targeted therapy treatment in VSCC are currently too limited to determine whether the molecular subtype can guide targeted therapy choices [31,58,64]. Evaluating biomarkers related to immune checkpoint therapy, Corey et al. [22] reported no significant differences in mismatch repair deficiency (dMMR/MSI-H), tumor mutational burden, and PD-L1 expression between the three different molecular subtypes.

Some results have shown that the *NOTCH* pathway may serve as a potential therapeutic target [38] through its inhibition by sarco-endoplasmic reticulum Ca^2+^-ATPase (SERCA) modulators [65]. The recently described third molecular pathway of VSCC may, therefore, be targetable by SERCA modulators. Additionally, *HRAS* mutations are prevalent in the molecular pathway of HPVi p53wt VSCC [37]. *HRAS* is involved in the RTK/RAS/PIK3-CA pathway and is targetable by selective mTOR inhibitors [66]. Treatment recommendations based on molecular subtype are not in the most recent update of the guidelines of the European Society of Gynaecological Oncology for the management of patients with vulvar cancer [31]; for this to happen, the molecular subtyping of VSCC will first have to be incorporated into future studies.

## 8. Conclusions

**Figure 6:**
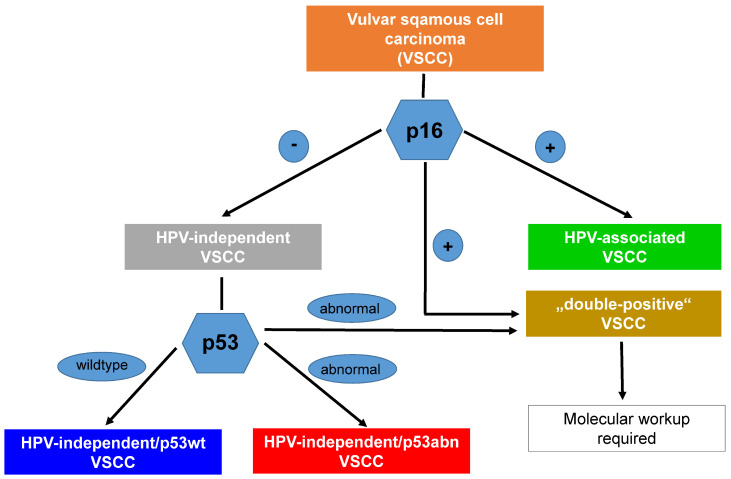
Algorithmic approach in routine pathology workup for the three-tiered molecular classification of VSCC [10,14,40].

**Figure 7:**
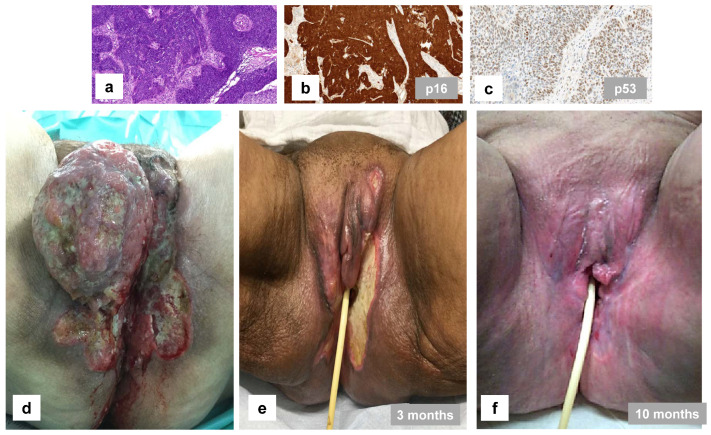
A 57-year-old woman with a poorly differentiated squamous cell carcinoma ((**a**); FIGO stage IVA) with p16 block-type staining overexpression (**b**) and p53 wild-type expression staining pattern (scattered; (**c**)) on immunohistochemistry, treated using hypofractionated radiation with a total dose of 38 Gy and sequential chemotherapy consisting of 5 cycles of carboplatin AUC 5 and paclitaxel 175 mg/m^2^. (**d**) Prior treatment: 25 cm × 10 cm exophytic superficially ulcerated tumor. (**e**) Partial clinical response after three months of treatment. (**f**) Complete local clinical response after 10 months. There was no local recurrence within 363 months of follow-up.

**Figure 8:**
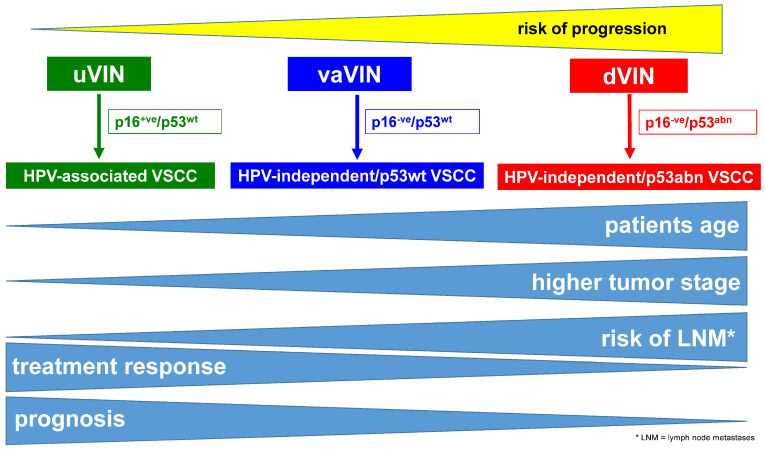
Summary of the three-tiered molecular classification of VSCC.

In more than 95% of cases, VSCC can be reliably subclassified into one of three different molecular subtypes using immunohistochemistry alone [14,40,50]. Immunostaining for both p16 and p53 is required for molecular subtype diagnosis [14,17], and attention to recent improvements in the interpretation of both of these immunomarkers is critical (see Table 1 and Figure 5 and Figure 6) [40,41,67,68]. While the molecular subtypes of VSCC are of prognostic significance, more data are needed to assess whether they can guide the extent of surgery or choice of adjuvant therapy, as is the case with the molecular classification of other tumor types, such as breast and endometrial carcinoma. The generation of such data will be facilitated by a routine and accurate molecular classification of all VSCCs; this is particularly critical for cases included in research studies/clinical trials. The impact of the three-tiered molecular subclassification of VSCC is schematically summarized in Figure 8.

## Figures and Tables

**Table 1:** Pattern-based analysis of p53-immunoexpression in VSCC for molecular classification ([22,32,40]; please see text).

**(1)** **p53-staining patterns in HPV-high risk related VSCC with p16-overexpression**		
*scattered pattern* patchy positivity mostly within basal layers, with heterogeneous nuclear staining intensity	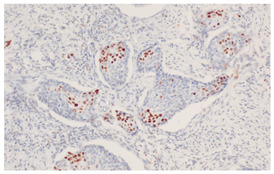	
*basal sparing/mid-epithelial* nuclear negativity of the outer/basal cell layers of the invasive growing tumor cell nest, often accompanied by increased staining in the mid and central/suprabasal cell layers	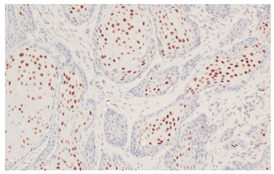	
**(2)** **p53-staining patterns in HPV-high risk negative VSCC without p16-overexpression**		
**staining pattern**		**mutational background**
*basal overexpression* strong, homogeneous nuclear staining of ≥80% of basal cells without significant staining of suprabasal cells within the invasive growing tumor cell nest	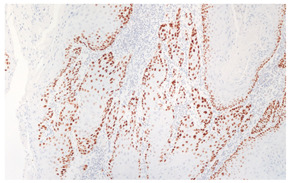	Missense mutation
*parabasal/diffuse overexpression* strong, homogeneous nuclear staining of ≥80% of basal cells and of suprabasal cells within the invasive growing tumor cell nest	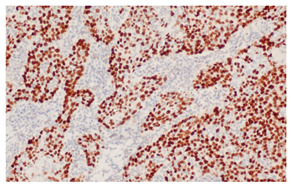	Missense mutation
*absent/null staining* complete negative staining of the lesional cells with positive nuclear staining of adjacent stromal cells/lymphocytes, i.e., internal positive control	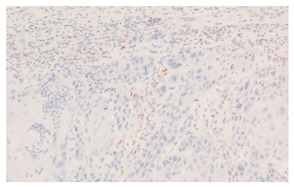	Nonsense or frameshift mutation
*cytoplasmic positivity* clearly visible, most granular staining of the cytoplasm of the lesional cells with variable nuclear staining	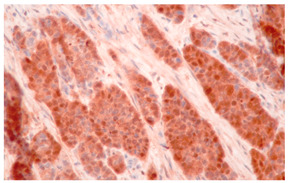	Mutation affecting the nuclear transportation of the p53-protein

**Table 2:** Frequency of the different molecular subtypes reported in the literature.

	Case Number	Treatment	HPV-Associated	HPV-Independend/p53 mut	HPV-Independend/p53 wt
Horne et al. (2018) * [53]	73	RX/RCX	38.3%	31.5%	23.3%
Barlow et al. (2020) [15]	119	OP + adj. RX	52.9%	37.0%	10.1%
Kortekaas et al. (2020a) [12]	413	OP + adj. RCX	18.1%	66.6%	15.2%
Woelber et al. (2021a) [39]	411	mixed	32.1%	39.7%	28.2%
Thompson et al. (2022) [20]	68	VFR	32.3%	60.3%	7.4%
Corey et al. (2023) [22]	105	N.A.	37.1%	49.5%	13.4%
Carreras-Dieguez et al. (2023a) [21]	190	OP + adj. R(C)X	17.4%	72.1%	10.5%
**pooled**	**1.379**		**32.6%**	**50.9%**	**15.4%**

* p16+/p53+: 5/73 = 6.8%; N.A. = not applicated.

**Table 3:** Clinicopathologic characteristics of the *three-tiered* molecular classification of VSCC [12,39].

	HPV-Associated	HPV-Independent/p53 Wildtype	HPV-Independent/p53 Abnormal	*p*-Value
**Frequency**				
Kortekaas et al. (2020a) [12]	18.1% (75/413)	15.2% (63/413)	66.7% (275/413)	
Woelber et al. (2021a) [39]	32.1% (132/411)	28.2% (116/411)	39.7% (163/411)	
**Age**				
Kortekaas et al. (2020a) [12]—median	59 years	73 years	75 years	*p* < 0.0001
Woelber et al. (2021a) [39]—mean	63.5 years	68.9 years	71.6 years	*p* < 0.001
**Tumor stage**				
Kortekaas et al. (2020a) [12]—FIGO ≥ II	21.30%	22.20%	47.30%	*p* < 0.0001
Woelber et al. (2021a) [39]—pT1b	33.30%	28.40%	23.90%	*p* = 0.15
**Tumor Size**				
Kortekaas et al. (2020a) [12]				
median	1.9 cm	2.5 cm	2.5 cm	*p* = 0.25
≤2 cm	52.00%	38.10%	36.40%	N.A.
Woelber et al. (2021a) [39]				
mean	3.9 cm	3.5 cm	3.4 cm	*p* = 0.51
**Depth of invasion**				
Kortekaas et al. (2020a) [12]—median	3.0 mm	4.5 mm	6.0 mm	*p* = 0.001
Woelber et al. (2021a) [39]—mean	6.9 mm	8.4 mm	9.7 mm	*p* = 0.13
**LVSI**				
Kortekaas et al. (2020a) [12]	9.30%	9.50%	16.00%	*p* = 0.03
**Nodal status**				
Woelber et al. (2021a) [39]—pN+	28.00%	30.20%	42.30%	*p* = 0.050

**Table 4:** Prognostic impact of the three-tiered molecular classification of VSCC [11,12,18,39,63].

	Outcome		HPV-Associated	HPV-Independent		
	Variables		(p16^+ve^/p53^wt^)	(p16^−ve^/p53^abn^)	(p16^−ve^/p53^wt^)	*p*-Value
Nooji et al. (2017) [37]	N = 236		N = 38 (16.1%)	N = 155 (65.7%)	N = 43 (18.2%)	
		**recurrence ≤ 2 yrs**	5.30%	22.60%	16.30%	*p* = 0.044 *
		**overall survival**	75.00%	67.20%	56.30%	*p* = 0.296
		**D.O.D.**	21.60%	45.90%	39.50%	*p* = 0.043
Hinten et al. (2018) [11]	N = 347		N = 55 (15.5%)	N = 263 (75.8%)	N = 29 (8.4%)	
		**recurency rate**	20%	36%	21%	*p* = 0.024
		**D.O.D.**	12.70%	34.20%	31.00%	NA
Kortekaas et al. (2020a) [12]	N = 413		N = 75 (18.1)	N = 275 (66.6%)	N = 63 (15.3%)	
		**recurrency rate**	14.70%	43.30%	25.40%	*p* < 0.0001
		**D.O.D.**	18.70%	65.10%	46.90%	*p* < 0.001
Barlow et al. (2020) [18]	N = 119		N = 63 (52.9%)	N = 44 (37.0%)	N = 12 (10.1%)	
		**disease specific**	89%	75%	83%	NA
		**survival**				
Woelber et al. (2021) [39]	N = 411		N = 132 (32.1%)	N = 163 (39.7%)	N = 116 (28.2%)	
		**recurrent disease**	22.70%	34.90%	30.20%	NA
		**2-year disease free**				
		**Survival**	63.90%	47.10%	60.20%	*p* < 0.001
		**2-year overall**				
		**Survival**	82.50%	70.40%	75.40%	*p* = 0.002

D.O.D. = died of the disease, NA = not applicated. * No significant difference between the local recurrence rate by comparing p53mut and HPV/p16-/p53wt cases (*p* = 0.246).
